# Dramatic, durable response to therapy in g*BRCA2*-mutated pancreas neuroendocrine carcinoma: opportunity and challenge

**DOI:** 10.1038/s41698-023-00376-x

**Published:** 2023-04-22

**Authors:** Fergus Keane, Raazi Bajwa, Pier Selenica, Wungki Park, Michael H. Roehrl, Jorge S. Reis-Filho, Diana Mandelker, Eileen M. O’Reilly

**Affiliations:** 1 Department of Medicine, Memorial Sloan Kettering Cancer Center, New York, NY USA; 2 David M. Rubenstein Center for Pancreatic Cancer Research, New York, NY USA; 3 Department of Radiology, Memorial Sloan Kettering Cancer Center, New York, NY USA; 4 Department of Pathology and Laboratory Medicine, Memorial Sloan Kettering Cancer Center, New York, NY USA; 5 Department of Medicine, Weill Cornell Medical College, New York, NY USA; 6 Parker Institute for Cancer Immunotherapy, San Francisco, CA USA; 7 Diagnostic Molecular Genetics Laboratory, Memorial Sloan Kettering Cancer Center, New York, NY USA

**Keywords:** Pancreatic cancer, Pancreatic cancer

## Abstract

Poorly differentiated pancreatic neuroendocrine tumors (PDNEC), are a subtype of pancreatic cancer encompassing both small cell and large cell neuroendocrine carcinoma subtypes, and are characterized as distinct in terms of biology and prognosis compared to the more common pancreatic adenocarcinoma. Until recently, there has been a paucity of data on the genomic features of this cancer type. We describe a male patient diagnosed with PDNEC and extensive metastatic disease in the liver at diagnosis. Genomic analysis demonstrated a germline pathogenic variant in *BRCA2* with somatic loss-of-heterozygosity of the *BRCA2* wild-type allele. Following a favorable response to platinum-based chemotherapy (and the addition of immunotherapy), the patient received maintenance therapy with olaparib, which resulted in a further reduction on follow-up imaging (Fig. 1). After seventeen months of systemic control with olaparib, the patient developed symptomatic central nervous system metastases, which harboured a *BRCA2* reversion mutation. No other sites of disease progression were observed. Herein, we report an exceptional outcome through the incorporation of a personalized management approach for a patient with a pancreatic PDNEC, guided by comprehensive genomic sequencing.

## Introduction

Pancreatic neuroendocrine tumors (NETs) have an incidence of 1.8 per 1,000,000 in females and 2.6 per 1,000,000 in males, based on the National Cancer Institute’s Surveillance Epidemiology and End Results (SEER) program data^1^. The World Health Organization (WHO) characterizes PDNECs by a high mitotic count (>20 mitoses/2 mm^2^), and a high Ki-67 index (typically >55%)^2^. PDNECs are aggressive cancers, ofttimes diagnosed at advanced stage and portending poor outcome^3^. In this case, we highlight the value of a precision oncology approach to the management of patients with this rare cancer type, resulting in a uniquely favorable outcome for this patient.

## Results

### Case report

A 66-year-old male presented with a short history of escalating abdominal pain, distension, nausea, and fatigue. Physical examination was notable for an ill-appearing man with scleral icterus and hepatomegaly. Laboratory investigations demonstrated marked hepatic dysfunction, notably alanine aminotransferase 193 (<55 U/L), aspartate aminotransferase 123 (<37 U/L), alkaline phosphatase 238 (<130 U/L), total bilirubin 5.5 (<1.2 mg/dL), and lactate dehydrogenase 570 (130–250 U/L). His past medical history was unremarkable. Family history included carcinoma of unknown primary in his mother at age 60, prostate cancer in his father at age 85, and lung cancer in his brother at age 68. He was hospitalized for expedited evaluation and management.

**Fig. 1:**
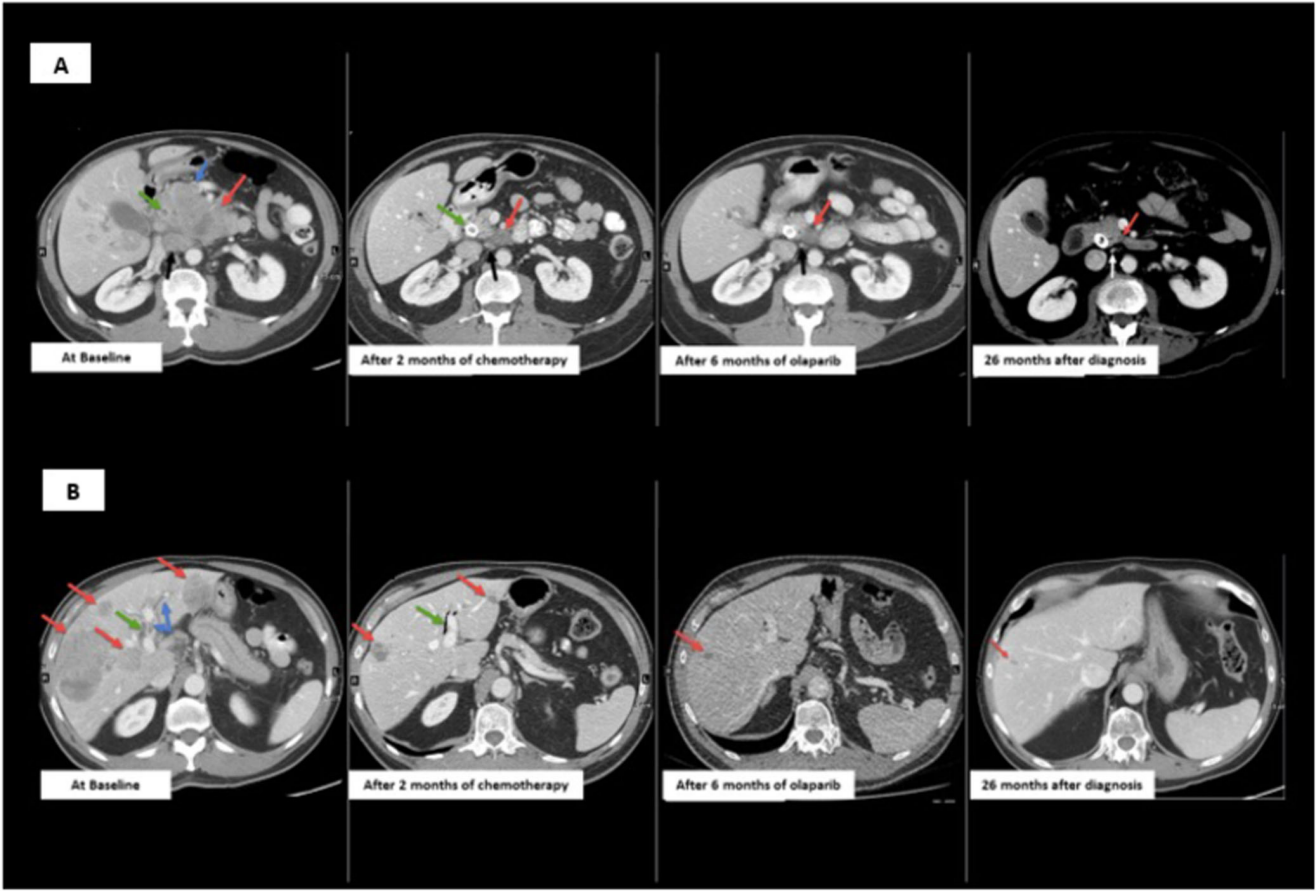
Computed Tomography (CT) demonstrating responses to therapy. **A** CT abdomen with contrast through the pancreas. At Baseline, pre-treatment CT shows pancreatic head mass (red arrow) invading the superior mesenteric vein (blue arrow) and obstructing the common bile duct (green arrow). Enlarged aortocaval adenopathy (black arrow). After 2 months of platinum-based chemotherapy, CT shows markedly decreased pancreatic tumor (red arrow) and aortocaval adenopathy (black arrow), and placement of metal common bile duct stent (green arrow). After 6 months of Olaparib, CT demonstrates further reduction in pancreatic tumor (red arrow) and aortocaval adenopathy (white arrow). 26 months after diagnosis, CT demonstrates ongoing response to PARPi in pancreatic tumor (red arrow) and aortocaval adenopathy (white arrow). **B** CT abdomen with contrast through the liver. At baseline, pre-treatment CT shows multiple bilobar liver metastases (red arrows), portal vein bland thrombus (blue arrows) and dilated intrahepatic bile duct (green arrow). After 2 months of platinum-based chemotherapy, CT shows resolved and markedly decreased liver metastases (red arrows), resolved portal vein thrombus and new pneumobilia (green arrow) from metal common bile duct stent placement (not shown). After 6 months of Olaparib, CT demonstrates further reduction of liver metastases (red arrow). 26 months after diagnosis, CT demonstrates ongoing response of liver metastases (red arrow) to PARPi. *PARPi poly (ADP-ribose) polymerase inhibitor.

**Fig. 2:**
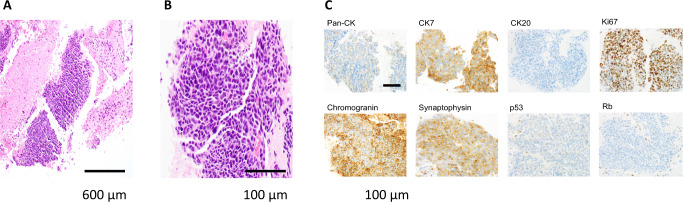
Histologic findings from diagnostic liver biopsy, demonstrating poorly differentiated neuroendocrine carcinoma. **A** Hematoxylin and Eosin (H&E), magnification 100×, scale bar: 600μm, **B** H&E magnification 600×, scale bar: 100 μm). The neoplastic cells expressed CK7, chromogranin and synaptophysin, aberrant loss of P53 and RB protein expression and a high Ki67 labeling index (**C** 3,3′-Diaminobenzidine, magnification 600×, scale bar: 100 μm).

Computed tomography (CT) demonstrated a mass in the head of the pancreas with invasion of the superior mesenteric vein, enlarged aortocaval adenopathy (Fig. 1A), bi-lobar liver metastases, intrahepatic biliary duct dilatation and portal vein bland thrombus (Fig. 1B). An endoscopic retrograde cholangiopancreatography (ERCP) was performed, and common bile duct and pancreatic stents were inserted. A liver biopsy demonstrated a poorly differentiated carcinoma, with tumor cells positive for cytokeratin (CK) 7, synaptophysin and chromogranin, aberrant loss of P53 and RB protein expression, and a Ki67 proliferation index of 90%, consistent with a diagnosis of PDNEC carcinoma of pancreas origin, with no adenocarcinoma component (Fig. 2A–C).

The patient initiated chemotherapy with cisplatin and etoposide, administered day 1–3 every 21 days. Within two weeks of receiving the first cycle, he had experienced marked symptomatic improvement. Liver function tests improved. Given the patient’s diagnosis at age 66, and a family history including a cancer diagnosis in his mother at age 60, he consented to undergo germline testing for the presence of a pathogenic variant. A germline pathogenic variant in *BRCA2* was identified, and loss-of-heterozygosity (LOH) of the *BRCA2* wild-type allele was detected on somatic sequencing of the liver metastasis. A restaging computerized tomography (CT) scan performed after three cycles of treatment demonstrated a dramatic response to chemotherapy in all disease sites (Fig. 1A, B). The patient continued platinum/etoposide chemotherapy for three additional cycles, and while not routine in extrapulmonary PDNEC, extrapolating from data in small cell lung cancer demonstrating a survival benefit with the addition of immune checkpoint blockade to platinum-based chemotherapy, atezolizumab was added for three cycles (a total of six cycles of cisplatin and etoposide, with the addition of atezolizumab for the latter three cycles), following which further radiologic responses in both the pancreas primary and the liver metastases, were observed.

Owing to cumulative toxicity with fatigue and emerging peripheral neuropathy, chemotherapy and immunotherapy were discontinued. The poly (ADP-ribose) polymerase inhibitor (PARPi) olaparib was commenced based on the *BRCA*2 mutation, extrapolating directly from the data supporting the use of PARPis in the post-platinum, maintenance setting for patients with pancreatic adenocarcinoma^4^, ovarian cancer^5^, breast cancer^6,7^, and prostate cancer^8^ harbouring germline *BRCA* pathogenic variants. Follow-up CT scans indicated further tumor response and ongoing disease control with olaparib (Fig. 1A, B), and the patient maintained an excellent quality of life (QoL). After 17 months, he developed left upper extremity weakness, poor co-ordination and falls. A right parietal lobe enhancing lesion with surrounding vasogenic edema was identified on Magnetic Resonance Imaging (MRI), consistent with a metastasis (Supplementary Fig. 1). No extracranial disease progression was identified on restaging imaging. Following multidisciplinary discussion, the patient underwent resection of this lesion, and histology was consistent with a metastasis from the PDNEC. He received post-operative stereotactic radiation to the tumor bed. Within eight weeks of completion of radiation, he developed new neurologic symptoms and dural-based parietal metastases were identified on MR imaging. He underwent a second craniotomy, followed by radiotherapy, but despite these interventions intracranial disease progression continued, without systemic relapse (Fig. 1A, B). The patient passed away 26 months after initial diagnosis.

### Genomic analysis

**Fig. 3:**
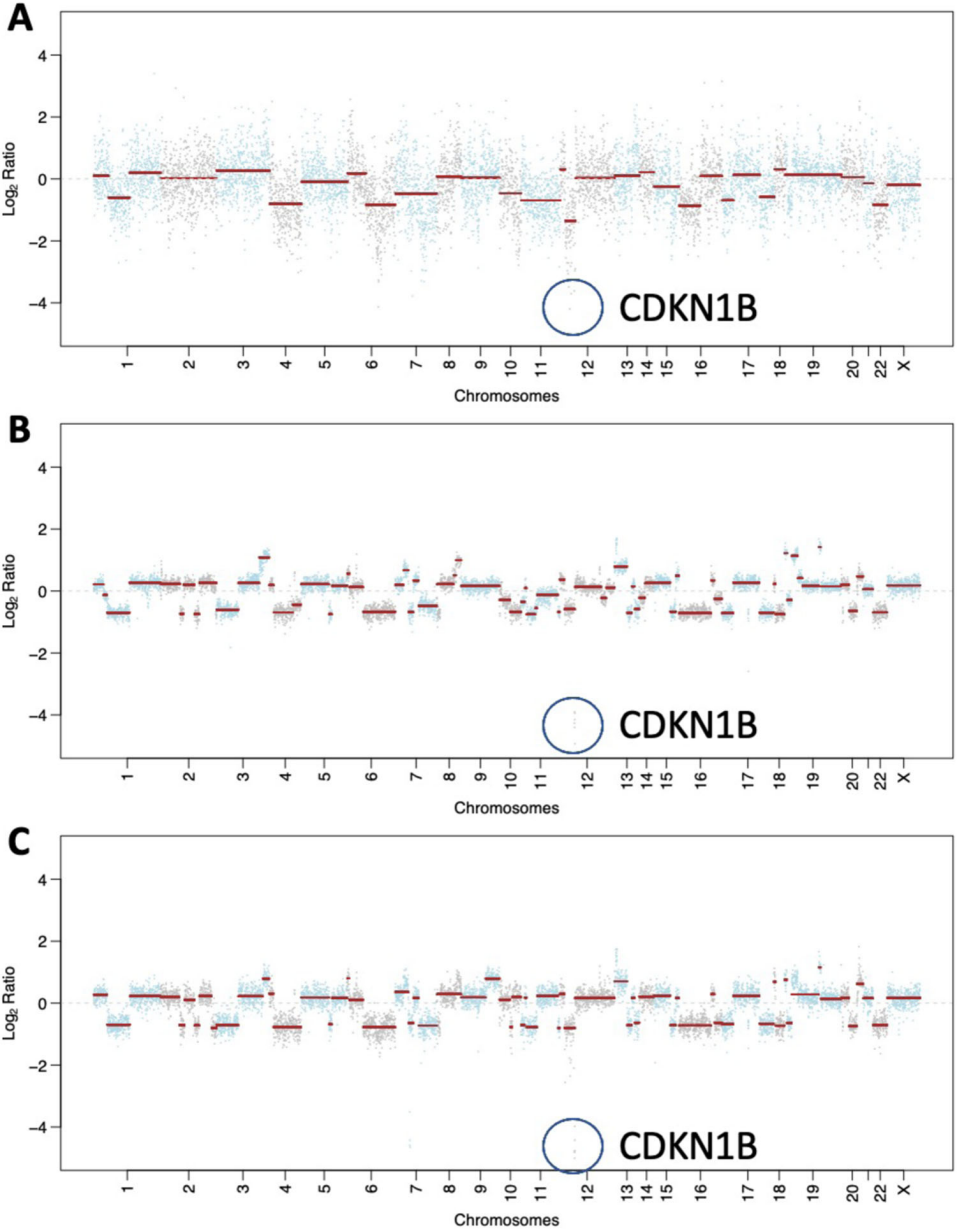
Somatic mutations and copy number alterations called from MSK-IMPACT targeted panel sequencing. **A** Copy Number Plot of pre-treatment liver metastasis. **B** Copy Number Plot of post-chemotherapy and olaparib resected CNS metastasis. **C** Copy Number Plots of post-chemotherapy, Olaparib, and radiotherapy CNS metastasis. In **A**–**C**, the genomic locus of *CDKN1B* is highlighted by a blue circumference.

Germline and somatic genomic testing were carried out utilizing the FDA-authorized Memorial Sloan Kettering Integrated Mutation Profiling of Actionable Cancer Targets (MSK-IMPACT), at baseline for this patient, prior to institution of systemic therapy. In addition to *BRCA2* biallelic loss-of-function and consistent with a diagnosis of PDNEC, the tumor harboured biallelic loss-of-function alterations affecting *TP53* and *RB1*, a *CDKN1B* homozygous deletion, and a G12R *KRAS* hotspot mutation (Fig. 3). Although *KRAS* and *TP53* alterations can be associated with pancreatic ductal adenocarcinoma, collectively the genomic profile, as well as the histopathologic findings (Fig. 2) were consistent with a bona fide high grade PDNEC harboring homologous recombination deficiency.

**Fig. 4:**
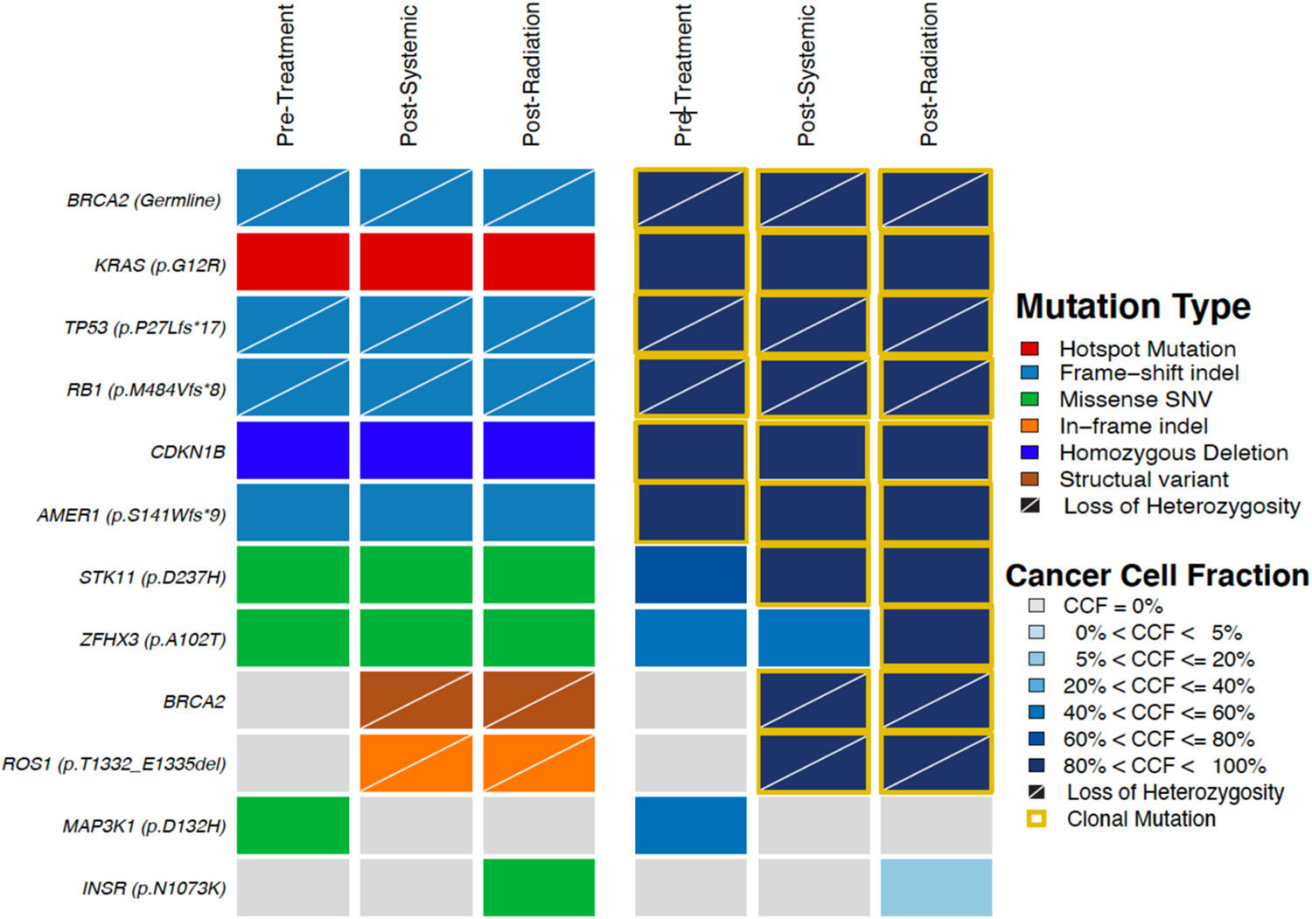
Heatmap demonstrating identified genomic alterations in each sample (pre-treatment liver metastasis, post chemotherapy and olaparib CNS metastasis, and post systemic therapy and radiation CNS metastasis, as labeled). The type of alteration is indicated by the color coded heatmap on the left side of the panel and the cancer cell fraction (i.e the proportion of cancer cells harboring an alteration) of each alteration is indicated by the heatmap on the right side of the panel.

**Fig. 5:**
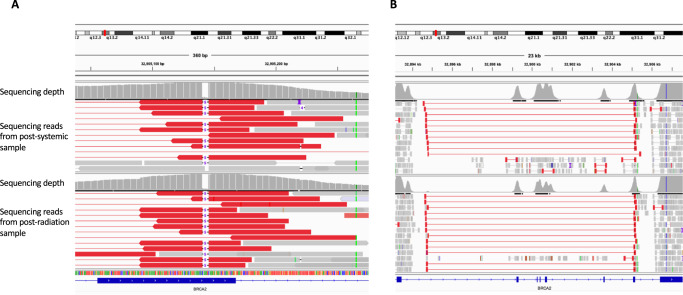
Integrative Genomics Viewer (IGV) screenshot showing the overlap between the reversion structural variant and the germline mutation in *BRCA2*. Thin red lines show the length of the reads supporting the structural variant and thick red lines show the ends of the reads delineating the breakpoints of the structural variant. The thin black lines with a “5” in the middle demonstrate the germline 5 base pair deletion. The histograms above each sample’s reads show the depth of sequencing at that locus. **A** IGV screenshot showing the overlap between the end of the reversion structural variant and the germline short deletion in the post-systemic therapy sample (top) and post-radiation sample (bottom). **B** IGV screenshot showing the region of *BRCA2* affected by the structural variant in the post-systemic therapy sample (top) and post-radiation sample (bottom).

Comprehensive tumor sequencing of both resected CNS metastases were compared with the sequencing results from the baseline diagnostic liver biopsy (Fig. 3). Both brain metastasis specimens demonstrated the presence of a *BRCA2* structural rearrangement spanning the locus of the *BRCA2* germline pathogenic alteration, which was not identified in the pre-treatment specimen. This rearrangement removed the stop codon caused by this pathogenic germline mutation (Fig. 4), and likely resulted in restoration of homologous recombination DNA repair and resistance to olaparib. Although a similar spectrum of genomic alterations was observed in the two resected CNS specimens, the apparent emergence of different clones was noted, although these specimens were only 12 weeks apart (Fig. 5).

Cell free DNA (ctDNA) was assessed at the time of second intracranial disease recurrence, identifying an *ATM* p.L695Wfs*s alteration not detectable in the tumor specimens and with a low variant allele fraction of 0.28%, consistent with clonal hematopoiesis of indeterminate potential^9^.

## Discussion

The first-line treatment of PDNECs has traditionally followed that of small cell lung cancer, with best evidence supporting the use of cisplatin and etoposide^10,11^. A diagnosis of PDNEC portends a poor prognosis, with a median survival of 5.7 months^12^. The incorporation of a maintenance phase of therapy into treatment paradigms is a subject of investigation across cancer types and is aimed to maintain and/or induce disease regression, while typically allowing de-escalation or removal of cytotoxic therapy to reduce toxicity and maximize QoL. Notable, relevant examples pertinent to this case are in the setting of extensive stage small cell lung cancer, where international guidelines incorporate immune checkpoint inhibitors, atezolizumab or durvalumab, in combination with chemotherapy, followed by maintenance checkpoint blockade without cytotoxic therapy until disease progression, based on two FDA approvals^13^. Additionally, the phase III Pancreas Olaparib Ongoing (POLO) trial elucidated a biomarker driven approach to the treatment of advanced pancreas ductal adenocarcinoma (PDAC) and marked a potential new era of precision medicine for this disease^4^ with a progression free survival advantage over placebo observed for maintenance olaparib in patients with germline *BRCA1/2* mutated PDAC and platinum-sensitive disease. Benefits have been observed across tumor types, including ovarian^5^, breast^6,7^ and prostate cancer^8^, leading to regulatory approvals of PARPis in these diseases.

Somatic *BRCA2* LOH was detected in the tumor samples from this patient. Previous work from our group has indicated that in PDAC, patients with biallelic alterations affecting homologous recombination DNA repair-related genes (e.g., *BRCA1/2*) are the most likely to benefit from platinum-based therapy^14,15^ and PARPi therapy^14^, with often durable responses. Those with biallelic status, such as described herein, are associated with a higher TMB, and display a COSMIC Signature 3, which are indicators of greater genomic instability, and postulated to be more likely to benefit from immunotherapy^16,17^ in the context of *BRCA2* alterations^18^. In view of the excellent response to platinum-based chemotherapy, and extrapolating from the above data with a goal to personalize treatment recommendations, we opted to proceed with maintenance PARPi therapy, which resulted in a prolonged period of systemic disease control. Given the initial dramatic response to platinum-based chemotherapy (and immunotherapy) observed, it is possible that the disease responses identified on subsequent imaging were due entirely to ongoing, exceptional response to chemotherapy, and not secondary to olaparib. A recent series from Symonds et al.^19^ demonstrated remarkable disease control with olaparib in a patient with de novo PDNEC of the prostate with *BRCA2* loss, again in the post-platinum chemotherapy setting. In our case, as in most scenarios, the commencement of PARPi was the setting of disease response to platinum-based therapy, thus definitively determining which agent has resulted in disease response and control, can prove challenging.

The case herein demonstrates significant benefits arising from results of genomic testing for this patient. Current international guidelines including the National Comprehensive Cancer Network (NCCN) guidelines, recommend that genetic counseling and testing for inherited genetic conditions should be considered, but do not recommend universal genomic testing for all patients with neuroendocrine tumors, including PDNEC^17^. While data pertaining to comprehensive genomic sequencing in pancreatic NECs has traditionally been sparse, a number of recent series have demonstrated enrichment for alterations in *TP53*, *RB1*, *APC*, *KRAS*, *BRAF*^18,19^, copy number losses in *ARID1A, ATM* and *ESR1*, in addition to amplifications/gains in *MYC* and *KDM5A*^19^. Recent studies in both pancreatic and non-pancreatic gastrointestinal NECs have demonstrated the presence of potentially actionable alterations^19,20^. Specific to *BRCA* alterations, one series demonstrated that 25% of patients with neuroendocrine cancer of the prostate had detectable biallelic *BRCA2* alterations^16^, underpinning a critical need to recommend genomic testing to detect potentially actionable targets and is applicable across solid tumor types. Given this data, as well as data in *BRCA* mutated pancreatic adenocarcinoma demonstrating the predictive implications of biallelic status^14^, in the setting of the identification of a somatic *BRCA* alteration we would suggest consideration of both reflexive germline testing as well as adjudication of biallelic/monoallelic status. The potential of comprehensive genomic testing to offer meaningful therapeutic benefits to patients may be underappreciated in the context of rare cancer types, where a paucity of genomic characterization has been conducted.

While the initial rationale behind the use of atezolizumab in this case was based on extrapolation from the survival data in small cell lung cancer^8^, the presence of the *BRCA2* alteration further supported its use. In our previous series of patients with PDAC and mutations in genes associated with homologous recombination deficiency (HRd) e.g. *BRCA 1/2*, those with biallelic status such as described herein, were associated with a higher TMB compared with patients with monoallelic status, or HRd wildtype status, and additionally display a COSMIC Signature 3, indicating greater genomic instability, and postulated to be more likely to benefit from immunotherapy^21,22^ in the context of *BRCA2* alterations^23^. In this instance, due to the limited genomic area covered in MSK-IMPACT targeted panel sequencing there were an insufficient number single nucleotide variants to compute single base substitution COSMIC mutational signatures. The optimal TMB at which immune checkpoint blockade is of benefit in neuroendocrine tumors remains an open question in the absence of randomized data, however FDA approval for the use pembrolizumab for tumors with TMB > 10 mutations per megabase (Muts/Mb) based on KEYNOTE-158 did include patients with metastatic neuroendocrine tumors^24^. In the case described herein, a TMB of 6.2 Muts/Mb was observed.

At 22 months from diagnosis, our patient developed symptomatic intracranial disease involvement, and despite best efforts with multi-modality approaches, intracranial disease control was not achieved. Prophylactic cranial irradiation (PCI) is not routinely recommended in major international guidelines for extra-pulmonary neuroendocrine carcinomas, in contrast to extensive stage small cell lung cancer, where PCI is considered based on an EORTC trial which demonstrated a survival advantage^25^. Our case is reflective of the challenge of intracranial disease progression, which can limit survival in patients with otherwise excellent systemic disease control. In the absence of randomized data, our case raises the question of whether PCI should be considered in select patients in whom systemic disease control is maintained.

Multiple potential causative factors may have led to the development of CNS metastases in this case, and merit pause for consideration. First, in this case a baseline imaging study of the central nervous system to assess for the presence of brain metastases was not performed, thus it is possible that occult brain metastases were present at the time of diagnosis, although no intracranial abnormality was noted on MRI Brain six months following diagnosis. Second, the presence of *BRCA* mutations is associated with a higher prevalence of CNS metastases in breast and ovarian cancers^26^, although this may be accounted for by several potential factors, including enrichment of triple-negative subtype in *BRCA*-driven breast cancers, as well as favorable survival in these subgroups. Data pertaining to whether a biological predisposition to CNS pattern of metastasis in *BRCA*-mutated pancreatic cancer is lacking. Third, while PARPi have resulted in CNS activity in some pre-clinical models^27^, other studies have shown suboptimal ability of the PARPis rucaparib and talazoparib to cross the blood brain barrier^28,29^. This is thought to be in part due to presence of P-glycoprotein (P-GP/ABCB1) and Breast Cancer Resistance Protein (BCRP) which function as efflux pumps at the blood brain barrier and limit CNS penetration. Olaparib is also a substrate of these proteins^30^ and therefore the CNS may represent a sanctuary site in this setting. Fourth, and perhaps the most parsimonious conclusion for this patient, the development of a CNS metastasis after 22 months of sustained disease control is best accounted for by the development of a reversion mutation (*BRCA2* rearrangement: c.316 + 1367 c.717del, Figs. 3 and 4).

The development of reversion mutations to targeted therapy, including specifically in *BRCA1/2*, leading to resistance to the PARPi, have been described^31,32^, albeit not, to our knowledge, in the setting of isolated CNS disease with ongoing excellent systemic control^33–37^. Overcoming resistance to targeted therapy remains a formidable challenge. The addition of immunotherapy to PARPi therapy is under investigation in several malignancies (NCT04548752, NCT04493060, NCT0466740), with benefit observed, as a potential combination to address resistance^38,39^. In addition, the combination PARPis with DNA polymerase θ (Polθ, also known as POLQ) inhibitors^40^ and ataxia telangiectasia and Rad3-related (ATR) inhibitors^41^ may constitute a strategy to overcome resistance caused by reversion mutations.

In conclusion, here we report a unique case of a patient with a PDNEC and a *BRCA2* germline mutation who had a dramatic response to platinum-based chemotherapy, followed by a period of deep, durable disease control with a PARPi, and complicated ultimately by a late intracranial relapse owing to a reversion mutation. We describe how extensive tumor sequencing uncovered that the intracranial relapse likely resulted from the development of a reversion mutation in the setting of prolonged PARPi use. This case highlights the value of a precision therapy approach which can lead to uniquely favorable outcomes for select patients, and in this regard supports the consideration of comprehensive genomic profiling, including in the management of patients with rare tumor types, where guidelines do not necessarily recommend universal genomic testing. It also re-enforces that in rare malignancies, where there is a dearth of randomized data, extrapolating from treatment paradigms for other malignancies with shared genomic features, should be entertained. Baseline CNS imaging to assess for the presence of intracranial metastases, as well as PCI should be considered in the setting of systemic disease control for PDNEC. Finally, this case provides direct evidence for the value of sequential genomic analyses at the time of disease progression on targeted therapies, to evaluate mechanisms of resistance.

## Methods

### Patient approval

Verbal consent for the publication of this report was provided by the patient and his family, and it is documented in the medical record.

### Ethics approval

This Case Report was reviewed by the Memorial Sloan Kettering IRB & Privacy Board Leadership (EM O’Reilly recused) on 10/19/2022 and the work was deemed non-human subjects research and hence does not require IRB oversight.

### Materials

The liver metastasis sample was obtained by CT guided core biopsy, and both CNS metastases were resected specimens taken at the time of surgery. All tumor samples were reviewed by a histopathologist with expertise in pancreatic cancers at MSK, confirming the presence of tumor cellularity sufficient for analysis by hematoxylin and eosin staining from formalin-fixed paraffin-embedded (FFPE) blocks. For somatic genomic analysis of all samples, DNA was extracted and purified, and tumor and normal comprehensive multi-gene panel sequencing was conducted using the FDA-authorized and New York State Department of Health approved Memorial Sloan Kettering–Integrated Mutation Profiling of Actionable Cancer Targets (MSK-IMPACT), which we have previously described^42^. Patient consent was taken for completion of both somatic and germline genomic analyses under institutional protocol IRB 12-245. Somatic single-nucleotide variants (SNVs), short insertions and deletions (Indels) and genomic re-arrangements were retrieved from the cBioPortal for Cancer Genomics (http://cbioportal.org)^43^. Binary and alignment mapped (BAM) files were retrieved and copy number alterations (CNAs) including loss of heterozygosity (LOH) was computed using FACETs^44^. BAM files were manually inspected using Integrative Genomics Viewer (IGV)^45^ to show the *BRCA2* locus affected by the germline mutation and re-arrangement. A mutation was classified as biallelic if there was a mutation concurrent with LOH of the wild-type allele. The cancer cell fraction (CCF) of each alteration was inferred using ABSOLUTE (v1.0.6), with the ABSOLUTE solutions manually reviewed. Alterations were classified as clonal if the probability of the alteration being clonal was >0.5 or if the lower confidence interval (CI) was >0.9 as calculated by ABSOLUTE^46^.

### Reporting summary

Further information on research design is available in the Nature Research Reporting Summary linked to this article.

## Supplementary information

Supplementary Figure 1

REPORTING SUMMARY

## Data Availability

The datasets supporting findings described in this study are available within the manuscript. Any additional information required can be made available upon request to the corresponding author.
