# Supplementary material for: Dramatic, durable response to therapy in gBRCA2-mutated pancreas neuroendocrine carcinoma: opportunity and challenge
Source: NPJ Precis Oncol. 2023 Apr 22;7:40. doi: 10.1038/s41698-023-00376-x (PMC10122663; doi:10.1038/s41698-023-00376-x)
Supplement: Supplementary file 1 — Supplementary Figure 1 [file 41698_2023_376_MOESM1_ESM.docx]

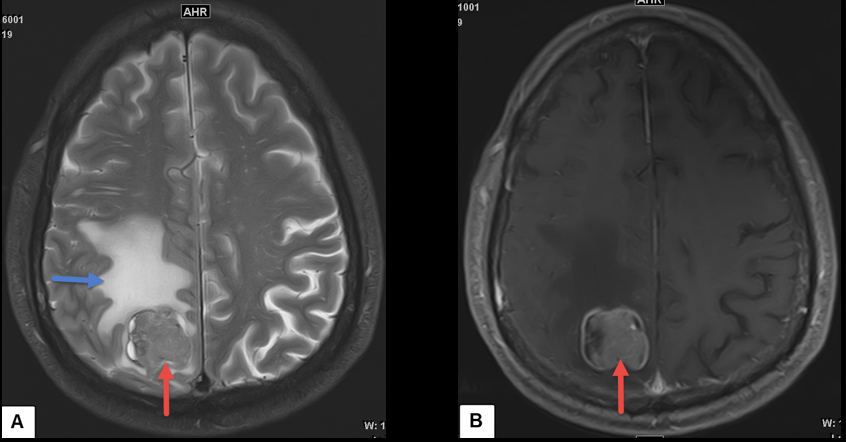


**Supplementary Figure 1.**

Magnetic Resonance (MR) Imaging of brain.

**A**. T2-weighted sequence shows a metastasis in the right parietal lobe (red arrow) with marked surrounding edema (blue arrow).

**B.** T1-weighted sequence post contrast shows marked enhancement of the metastasis (red arrow).
